# Second-Hand Smoke–Induced Cardiac Fibrosis Is Related to the Fas Death Receptor Apoptotic Pathway without Mitochondria-Dependent Pathway Involvement in Rats

**DOI:** 10.1289/ehp.7479

**Published:** 2005-06-01

**Authors:** Wei-Wen Kuo, Chieh-Hsi Wu, Shin-Da Lee, James A. Lin, Chia-Yih Chu, Jin-Ming Hwang, Kwo-Chang Ueng, Mu-Hsin Chang, Yu-Lan Yeh, Chau-Jong Wang, Jer-Yuh Liu, Chih-Yang Huang

**Affiliations:** 1 Department of Biological Science and Technology, China Medical University, Taichung, Taiwan; 2 Department of Physical Therapy, Chung-Shan Medical University, Taichung, Taiwan; 3 Department of Veterinary Medicine, National Chung-Hsing University, Taichung, Taiwan; 4 School of Applied Chemistry, and; 5 Department of Internal Medicine, Chung-Shan Medical University, Taichung, Taiwan; 6 Division of Cardiology, Armed Forces Taichung General Hospital, Taichung, Taiwan; 7 Department of Pathology, Changhua Christian Hospital, Changhua, Taiwan; 8 Institute of Biochemistry and Biotechnology, Chung-Shan Medical University, Taichung, Taiwan

**Keywords:** cardiac survival IGF-1 signaling, caspases, death-receptor-dependent pathway, mitochondria-dependent pathway, second-hand smoke (SHS)

## Abstract

Exposure to environmental tobacco smoke has been epidemiologically linked to heart disease among nonsmokers. However, the molecular mechanism behind the pathogenesis of cardiac disease is unknown. In this study, we found that Wistar rats, exposed to tobacco cigarette smoke at doses of 5, 10, or 15 cigarettes for 30 min twice a day for 1 month, had a dose-dependently reduced heart weight to body weight ratio and enhanced interstitial fibrosis as identified by histopathologic analysis. The mRNA and activity of matrix metalloprotease-2 (MMP-2), representing the progress of cardiac remodeling, were also elevated in the heart. In addition, we used reverse-transcriptase polymerase chain reaction and Western blotting to demonstrate significantly increased levels of the apoptotic effecter caspase-3 in treated animal hearts. Dose-dependently elevated mRNA and protein levels of Fas, and promoted apoptotic initiator caspase-8 (active form), a molecule of a death-receptor–dependent pathway, coupled with unaltered or decreased levels of cytosolic cytochrome *c* and the apoptotic initiator caspase-9 (active form), molecules of mitochondria-dependent pathways, may be indicative of cardiac apoptosis, which is Fas death-receptor apoptotic-signaling dependent, but not mitochondria pathway dependent in rats exposed to second-hand smoke (SHS). With regard to the regulation of survival pathway, using dot blotting, we found cardiac insulin-like growth factor-1 (IGF-1) and IGF-1 receptor mRNA levels to be significantly increased, indicating that compensative effects of IGF-1 survival signaling could occur. In conclusion, we found that the effects of SHS on cardiomyocyte are mediated by the Fas death-receptor–dependent apoptotic pathway and might be related to the epidemiologic incidence of cardiac disease of SHS-exposed non-smokers.

Second-hand smoke (SHS), a mixture of smog produced from a burning cigarette and exhaled smoke, contains thousands of chemical constituents, most of which are harmful and cause human diseases such as lung cancer and cardiovascular disease (Boyle and Maisonneuve 1995; Bronnum-Hansen 2000; De Cesaris et al. 1992; Haugen 2002; MacDougall et al. 1983). However, most studies on this subject focus on the association of SHS with respiratory symptoms, impaired lung function, and increased bronchial responsiveness. Actually, epidemiologic studies have showed that exposure to SHS increases the incidence of cardiac disease 4-fold (Ciruzzi et al. 1998; Stefanadis et al. 1998), and the mortality of cardiac failure among passive smokers is 38% higher than in those who do not smoke or who are not exposed to SHS (Baud et al. 1991; Ecanow and Blake 1978). Pope et al. (2001) found that 2 hr of exposure to SHS significantly destroyed cardiac autonomic function, as evidenced by decrements in heart rate variability in 16 adult nonsmokers. However, so far the mechanisms behind cardiac disease in subjects exposed to SHS are poorly understood.

Apoptosis is a recognized mechanism for the elimination of redundant cells, although it may also inhibit cell proliferation. In fact, it has been suggested that apoptosis plays a critical role in the pathogenesis of human diseases, including cardiac disorders (Haunstetter and Izumo 1998). Apoptosis has been reported to contribute to the loss of cardiomyocytes, after which the collagen secreted by fibroblasts replaces the space of damaged cardiomyocytes in cardiomyopathy. Hence, the fibrosis following apoptosis is recognized as a predictor of adverse outcomes in subjects with cardiomyopathy (Kim and Iwao 2000; Narula et al. 1996). Therefore, the evaluation of the apoptosis and/or fibrosis process could be an excellent way of predicting the development of cardiomyopathy induced by SHS, although the specificity of the related signaling pathways involved in the development of apoptosis and/or fibrosis needs to be identified.

The induction of apoptosis is associated with the activation of aspartate-specific cysteine protease, including caspase-3 (Fernandes-Alnemri et al. 1994). Several studies have demonstrated that mitochondria may play an important role in apoptosis by releasing cytochrome *c* and activating caspase-9, which activates caspase-3 that is responsible for DNA-cleavage action (Liu et al. 1996; Reed and Paternostro 1999). In end-stage cardiomyopathy, cytosolic cytochrome *c* is also accumulated (Narula et al. 1999). In addition, the death-receptor–induced apoptotic pathway is reportedly involved in the pathogenesis of cardiac disease (Scheubel et al. 2002). This pathway is initiated by death-receptor agonists, including the Fas ligand. After ligand binding, Fas receptor oligomerization results in the activation of caspase-8, which is upstream of caspase-3, causing the activation of apoptosis (Haunstetter and Izumo 1998). Therefore, as a common component of apoptotic signaling, caspase-3 mediates both mitochondria-dependent and death-receptor–dependent apoptotic pathways. Furthermore, the down-regulation of a survival pathway is another possible factor in the promotion of apoptosis in cells. In cardiomyocytes, insulin-like growth factor-1 (IGF-1), the survival factor through which IGF-1 receptor (IGF-1R) activates the phosphatidylinositol-3 kinase/protein kinase B (Parrizas et al. 1997) and the Ras-Raf-MEK-ERK (Ras–Raf–mitogen-activated protein kinase–extracellular receptor kinase) pathways, should be taken into consideration for preventing myocyte apoptosis (Hoshijima et al. 1998). Particularly, activated PI3K enhances the phosphorylation of Akt (Simoncini et al. 2000), which in turn regulates the activity of Bad and BCl_2_ to control the apoptosis of cardiomyocytes (Campbell et al. 2001).

To understand whether the effects of SHS on rat hearts are mediated through activating apoptotic pathways, including mitochondria-dependent and Fas death-receptor–dependent signalings, or through suppressing survival pathways, we examined the cardiac levels of signaling proteins and gene expression in these pathways by performing reverse-transcriptase polymerase chain reaction (RT-PCR), Western blotting, or dot blotting. We used these results to explore the molecular mechanisms of the pathogenesis of cardiac disease induced by cigarette smoke.

## Materials and Methods

### Animal model and cigarette smoke exposures.

We purchased male Wistar rats (6 weeks of age; body weight, 120 ± 10 g) from National Science Council Animal Center (Taipei, Taiwan). The animals were housed six per cage in an environmentally controlled animal room, and water was provided *ad libitum*. All animals were handled according to the guidelines of the Taiwan Society for Laboratory Animals Sciences for the care and use of laboratory animals (Institute of Laboratory Animal Resources 1996). We divided 24 rats into four exposure groups. The rats were placed in whole-body exposure chambers and exposed to 0, 5, 10, or 15 cigarettes (New Paradise, Taiwan), representing control, low, medium, and high doses, respectively. Filtered air was introduced into the chamber at a low rate of 200 L/min. Rats were exposed to cigarette smoke for 30 min, twice a day, 6 days/week for 1 month. Room temperature was maintained at 22–25°C, and relative humidity was approximately 40%. After 1 month, rats were weighed and killed. After removal from the thorax, the hearts were cleaned with double-distilled water and dried before weighing. The left and right atria and the right ventricle were then removed, and the left ventricle was weighed. We calculated the ratios of total heart weight and left ventricle weight to body weight.

### Hematoxylin-eosin and Masson trichrome staining.

Hearts were fixed in formalin, embedded in paraffin, and sectioned. Slides were hydrated through a series of graded alcohols (100, 95, and 75%), 15 min each. The slides were then stained with hematoxylin and eosin (H&E) or Masson trichrome. After gently rinsing with water, slides were dehydrated through graded alcohols for 15 min each, cleared in xylene, and coverslipped. Photomicrographs were obtained using Zeiss Axiophot microscopes (Zeiss, Oberkochen, Germany).

### Tissue extraction.

Cardiac tissue extracts were obtained by homogenizing the left ventricle samples in phosphate-buffered saline (PBS; 0.14 M NaCl, 3 mM KCl, 1.4 mM KH_2_PO_4_, 14 mM K_2_HPO_4_) at a concentration of 1 mg tissue/10 μL PBS for 5 min. The homogenates were placed on ice for 10 min and then centrifuged at 12,000 rpm for 30 min. The supernatant was collected and stored at –70°C for further experiments.

### Protein contents.

We determined the protein content of cardiac tissue extract using the Bradford protein assay (Bradford 1976) using the protein-dye kit (Bio-Rad, Richmond, CA, USA). We used a commercially available bovine serum albumin (Sigma Chemical, St. Louis, MO, USA) as a standard. Changes in optical density were monitored at 595 nm.

### Zymography protease assay.

We mixed the cardiac tissue extracts (40 μg) thoroughly with a suitable volume of PBS buffer and 4 μL dye. We carried out gelatin zymography analysis by loading 20 μL of the extracts on 8% SDS-PAGE gels containing 0.1% gelatin and run by electrophoresis at 140 V for 2.5 hr. The gels were washed in a 2.5% Triton X-100 solution with shaking for 30 min and then incubated in 50 mL reaction buffer (40 mM Tris-HCl, pH 8.0; 10 mM CaCl_2_, 0.01% NaN_3_) at 37°C for 12 hr before staining with 0.25% Coomassie brilliant blue R-250 in 50% methanol and 10% acetic acid for 1 hr. Quantitative analysis was preformed after discoloring the stain in a destaining solution (10% acetic acid, 20% methanol) twice for 30 min.

### Electrophoresis and Western blot.

We prepared the tissue extract samples as described above. SDS-PAGE was carried out with 10% polyacrylamide gels. The samples were electrophoresed at 140 V for 3.5 hr and equilibrated for 15 min in 25 mM Tris-HCl, pH 8.3, containing 192 mM glycine and 20% (vol/vol) methanol. Electrophoresed proteins were transferred to nitrocellulose paper (Hybond-C Extra Supported, 0.45 μm; Amersham, Piscataway, NJ, USA) using a Hoefer Scientific Instruments Transphor unit (Hoefer Scientific, San Francisco, CA, USA) at 100 mA for 14 hr. We incubated nitrocellulose papers in blocking buffer for 2 hr at room temperature and then in blocking buffer containing 100 mM Tris-HCl, pH 7.5, 0.9% (wt/vol) NaCl, and 0.1% (vol/vol) fetal bovine serum for 2 hr at room temperature. Monoclonal antibodies (Santa Cruz Biotechnology, Santa Cruz, CA, USA) were diluted 1:200 in antibody binding buffer containing 100 mM Tris-HCl, pH 7.5, 0.9% (wt/vol) NaCl, 0.1% (vol/vol) Tween-20, and 1% (vol/vol) fetal bovine serum. Incubations were performed at room temperature for 3.5 hr. We washed the immunoblots three times in 50 mL blotting buffer for 10 min and then immersed in the second antibody solution containing alkaline phosphatase goat anti-rabbit IgG (Promega, Madison, WI, USA) for 1 hr and diluted 1,000-fold in binding buffer. The filters were then washed in blotting buffer for 10 min three times. Color development was presented in 20 mL of a mixture consisting of 7 mg nitro blue tetrazolium, 5 mg 5-bromo-4-chloro-3-indolyl-phosphate, 100 mM NaCl, and 5 mM MgCl_2_ in 100 mM Tris-HCl, pH 9.5. Signal intensity of Western blots was quantitated using a PhosphoImager (Kodak, Rochester, NY, USA).

### RNA extraction.

We extracted total RNA using the Ultraspec RNA Isolation System (Biotecx Laboratories, Inc., Houston, TX, USA) according to the manufacturer’s instructions. Each heart was thoroughly homogenized in 1 mL Ultraspec reagent/100 mg tissue using a Polytron homogenizer (Kinematica AG, Lucerne, Switzerland). The homogenates were washed twice with 70% ethanol by gentle vortexing. RNA precipitates were then collected by centrifugation at 12,000 × *g* and dried under vacuum for 5–10 min before dissolving in 50 μL diethylpyrocarbonate-treated water; precipitates were then incubated at 55–60°C for 10–15 min.

### RT-PCR.

Total RNA (1 μg) was reverse transcribed and then amplified (30 cycles) by PCR using a Super Script preamplification system for first-strand cDNA synthesis and Taq DNA polymerase (Life Technologies, GIBCO BRL, Rockville, MD, USA). RT-PCR products (45 μL) were separated on a 1.25% low-melting-point agarose gel (Life Technologies). Amplimers were synthesized based on cDNA sequences from GenBank (2004). We used the human *pHe7* gene as an internal standard.

### RNA dot blotting.

We used RNA dot blotting for the hybridization and detection of *IGF-1* and IGF-1R mRNAs as described previously (Huang et al. 1998). The corresponding digoxigenin-labeled antisense RNA probes were prepared from pGEM-1 containing a *Bam*H1–*Eco*R1 956-bp insert consisting of exon 3 and flanking intron sequences from the rat *IGF-1* gene, and the pTRI-IGFR-human transcription template containing a 236-bp cDNA fragment of the human *IGF-1R* gene spanning exons 8–7 (Ambion, Austin, TX, USA).

### Statistical analysis.

We compared the data between groups of animals using one-way analysis of variance. We used Fisher’s least significant difference test to determine differences. *p*-Values < 0.05 were taken as significant.

## Results

### Cardiomyopathic alteration of rats administered different doses of SHS.

**Figure 1:**
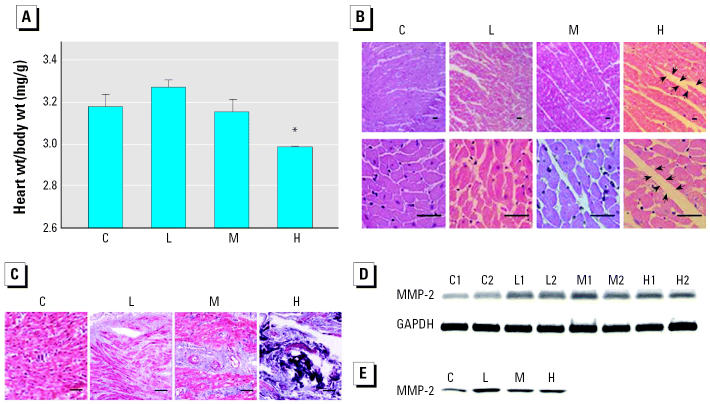
Cardiomyopathic changes in rats exposed to different doses of SHS. Abbreviations: C, control; H, high dose; L, low dose; M, medium dose; numbers beside the treatment represent the repetition. (*A*) The heart weight (wt) to body wt ratio (mean ± SD) of rats exposed to SHS (at least five rats per group). (*B*) Histopathologic analysis of cardiac tissue sections stained with H&E. Magnification: top, 100×; bottom, 400X; bars = 15 μm. Enlarged interstitium was observed in SHS-administered animal hearts, with the high-dose group showing the most significant changes; arrows indicate the myocardial interstitium. (*C*) Representative cross-sections with trichrome staining; fibrotic areas are stained blue. Bar = 15 μm; magnification, 200X. Extensive fibrosis and myofibril disarray are present in hearts from SHS-treated rats, particularly the high-dose group. Cardiac fibrosis induced by SHS is described in more detail in the “Discussion.” Alteration of MMP-2 levels in cardiac tissues as shown by MMP-2 mRNA levels analyzed by semiquantitative RT-PCR, using GAPDH as a loading control (*D*) and MMP-2 activities analyzed by zymography assay (*E*). *Significantly different compared with the control group (*p* < 0.05).

After 1-month administration of different doses SHS, all rats showed a generally healthy appearance. They were then weighed and killed, and the hearts were removed and weighed. We observed dark spots in the right ventricle of SHS-treated rats, and the ratio of whole heart weight to body weight showed significant reduction in the high-dose group (Figure 1A). To understand the possible cause of the decreased heart weight, we performed a histopathologic analysis of ventricular tissue stained with H&E as well as Masson trichrome. We found that the ventricular myocardium of healthy controls showed normal architecture and orderly alignment of myocytes with minimal interstitial fibrosis in tissues observed at both 100× and 400× magnification. In contrast, disarray with markedly enlarged interstitium of myocytes is evident in the SHS-administered group, and the most significant change was observed in the high-dose group (Figure 1B). Hearts from SHS-treated rats stained with Masson trichrome showed extensive fibrosis and myofibril disarray at the 200× magnification (Figure 1C). Masson trichrome staining also qualitatively revealed increased collagen deposition at minor and moderate levels in low-and medium-dose groups, respectively, but at a very strong level in the high-dose group. In addition, we evaluated the activity of matrix metalloprotease-2 (MMP-2), a gelatinase that can degrade extracellular matrix and that is associated with the morphologic changes. The treated groups had higher levels of both MMP-2 mRNA and protein activity than did the control group, although there was a decline in the high-dose group (Figure 1D,E).

### Changes of caspase-3 mRNA and active protein levels in the cardiac tissues of rats.

**Figure 2:**
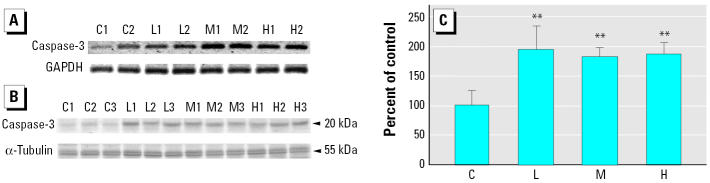
Activation of caspase-3 in cardiac tissues of rats. Abbreviations: C, control; H, high dose; L, low dose; M, medium dose; numbers beside the treatment represent the repetition. (*A*) Semiquantitative RT-PCR analysis of caspase-3 from left ventricles, using GAPDH as a loading control. (*B*) Western blot analysis of the activated form of caspase-3 from left ventricles, using α-tubulin as a loading control. (*C*) Caspase-3 shown as percent of control (mean ± SD of three independent experiments). **Significantly different compared with the control group (*p* < 0.01).

Exposure to SHS could lead to cardiomyocyte-apoptosis–related heart failure. In order to identify the promotion of apoptosis, we measured mRNA and the active protein levels of the executive apoptotic protein caspase-3. Both mRNA and the active protein levels of treated groups were higher than those of the controls (Figure 2).

### Alterations of component protein levels of Fas-receptor–dependent and mitochondria-dependent apoptotic pathways in the cardiac tissues of rats.

**Figure 3:**
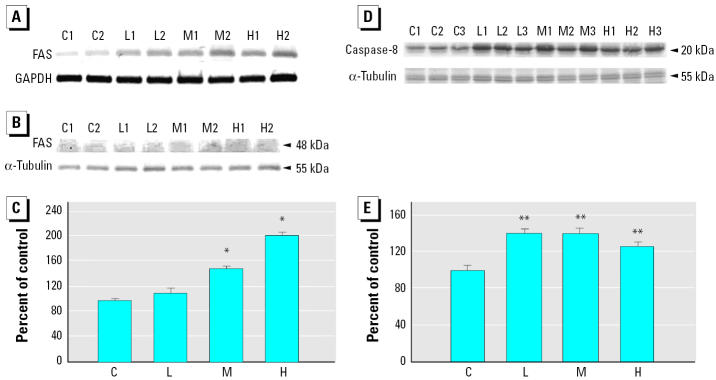
Activation of Fas and caspase-8 in cardiac tissues of rats. Abbreviations: C, control; H, high dose; L, low dose; M, medium dose; numbers beside the treatment represent the repetition. Fas activation in left ventricles shown by (*A*) semiquantitative RT-PCR analysis, (*B*) Western blot analysis, and (*C*) quantitation of signal intensity of FAS (mean ± SD of three independent experiments). Caspase-8 activation in left ventricles shown by (*D*) Western blot analysis and (*E*) quantitation of the signal intensity of the activated form of caspase-8 (mean ± SD of three independent experiments). *Significantly different compared with the control group (*p* < 0.05). **Significantly different compared with the control group (*p* < 0.01).

**Figure 4:**
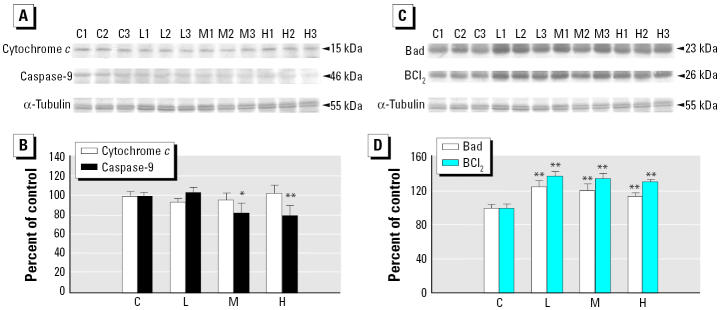
Activation of proteins related to apoptotic pathways in cardiac tissues. Abbreviations: C, control; H, high dose; L, low dose; M, medium dose; numbers beside the treatment represent the repetition. Cytosolic cytochrome c and the activated form of caspase-9 in left ventricles shown by (*A*) Western blot analysis and (*B*) quantitation of signal intensity of cytosolic cytochrome c and the activated form of caspase-9 (mean ± SD of three independent experiments). Bad and BCl_2_ in left ventricles shown by (*C*) Western blot analysis and (*D*) quantitation of the signal intensity of Bad and BCl_2_ (mean ± SD of three independent experiments). *Significantly different compared with the control group (*p* < 0.05). **Significantly different compared with the control group (*p* < 0.01).

To further understand the upstream signaling pathways associated with the activation of caspase-3, we examined the levels of the components of the Fas death-receptor–dependent and mitochondria-dependent/death-receptor–independent apoptotic pathways. Compared with control animals, both Fas mRNA and protein levels increased in a dose-dependent manner (Figure 3A–C). Additionally, all the treated groups showed significantly higher levels of active caspase-8 than did the control group (Figure D,E). Conversely, there was no difference in expression of cytosolic cytochrome *c* protein in any of the groups, and there was a significant decrease in active caspase-9 protein level in the medium- and high-dose groups (Figure 4A,B). Furthermore, both cardiac Bad and BCl_2_ levels were significantly higher in all treated groups, compared with the control group (Figure 4C,D).

### Induction of IGF-1 and IGF-1R mRNA in cardiac tissues of rats.

**Figure 5:**
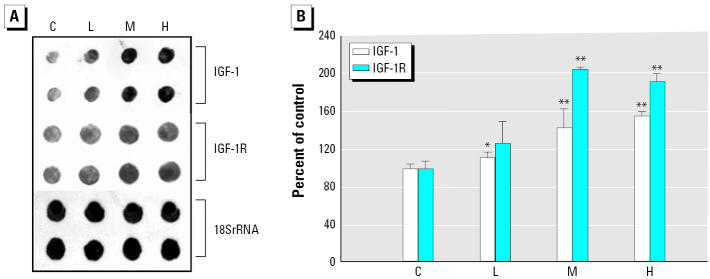
Induction of IGF-1 and IGF-1R gene expression in left ventricles from rats. Abbreviations: C, control; H, high dose; L, low dose; M, medium dose. (*A*) RNA dot blots prepared with 240 ng RNA/dot and probed with labeled oligonucleotide probes specific for the indicated genes. 18S RNA was used as a loading control. (*B*) Fold induction of gene expression (mean ± SD) for SHS-treated animals relative to control from three independent experiments. *Significantly different compared with the control group (*p* < 0.05).**Significantly different compared with the control group (*p* < 0.01).

We also examined cell survival. Except for the mRNA level of IGF-1R in the low-dose group, dot blotting analysis of cardiac survival factor showed mRNA of both IGF-1 and IGF-1R to have significantly greater increases than in the control group. This suggested that the survival pathway, IGF-1 signaling, might be up-regulated in cardiac tissue of rats exposed to SHS (Figure 5).

## Discussion

In the present study, after the exposure of 5, 10, or 15 cigarettes for 30 min, twice per day for 1 month, the rat hearts showed increased weight loss, interstitial fibrosis, and MMP-2 activity. After the confirmation of the activated caspase-3, a marker of apoptosis development, and the elevated levels of active caspase-8 and Fas protein, we did not observe increased levels of active caspase-9 or released cytochrome *c* in SHS-exposed rat hearts. This indicates that SHS-induced biologic cardiac responses associated with the activated caspase-3 may be involved with the Fas-signaling pathway. Furthermore, we demonstrated that cardiac expressions of IGF-1 and IGF-1R mRNA found by dot blotting were increased, suggesting that the compensatory effect of IGF-1 survival signaling also occurred in the hearts of the SHS-exposed rats.

Brilla et al. (1990) reported an association between the turnover of collagens and remodeling of the rat ventricles. The remodeling progresses immediately after myocardial damage with an increased level of collagenases (Janicki et al. 1995). Because MMP-2 is a member of gelatinase-A family, this enzyme is able to hydrolyze collagens I, IV, V, and VII (Dollery et al. 1995). Therefore, the level of MMP-2 might be predicted to increase during cardiac remodeling. Weber et al. (1992) reported that the severity of cardiac fibrosis may become significantly apparent with the development of remodeling by increasing MMP activity. That is, once the heart is damaged, extracellular matrix that connects cardiomyocytes will be degenerated by increased MMP for the adaptation of cardiomyocyte enlargement and fibroblast invasion. After hypertrophy, the dead apoptotic myocytes will be replaced by collagens, which are secreted by the invaded fibroblasts and will accumulate, resulting in ventricular fibrosis (Anversa and Nadal-Ginard 2002; Kim and Iwao 2000; Pacifico and Henry 2003; Takemura and Fujiwara 2003). The myocardial interstitial changes resulting from increased collagen deposition lead to cardiac stiffness and pathologic cardiac dysfunction (Anversa et al. 1996). Accordingly, the accumulated collagen, an extracellular matrix protein, will further contribute to the development of heart failure (Kim and Iwao 2000). In our study, the registered alignment of the normal myocardium characteristics became disordered by the administration of SHS (Figure 1B). Masson trichrome staining also showed extensive fibrosis and myofibril disarray in the SHS-exposed heart, with wider interstitial space and higher expression and activity levels of MMP-2 (Figure 1B–E). These results suggest the development of cardiac premature death characterized by the distortion in myocardium architecture and fibrosis. We believe that pathologic cardiac dysfunction can be predicted to occur if the experiment period is prolonged. Additionally, the data on IGF-1 signaling up-regulation in the present study might provide another explanation for the occurrence of cardiac fibrosis. The up-regulated IGF-1 signaling might promote the proliferation of noncardiomyocytes, such as fibroblasts, which may grow to fill in the space originally occupied by apoptotic cardiomyocytes, and again result in myocardial interstitial changes and cardiac fibrosis (Kim and Iwao 2000).

Apoptosis of cardiomyocytes has an important implication on cardiac dysfunction because of the reduced number of cardiomyocytes per functional units. The weight loss we found in smoking-induced rat ventricle in a dose-dependent manner (Figure 1A) could be associated with the progression of heart failure. The expression levels and activity increases of caspase-3 by RT-PCR and Western blotting (Figure 2) raise the possibility of cardiomyocyte apoptosis. Actually, caspase-3 is an important molecular marker of apoptotic signaling in that it modulates both mitochondria-dependent and Fas death-receptor–dependent apoptotic pathways. Further evidence confirming which of them is involved in the activation of caspase-3 is provided by the findings of elevated levels of caspase-8 and Fas, coupled with no alteration or even reduced levels of caspase-9 activity and cytosolic cytochrome *c* in ventricles of rats exposed to SHS (Figures 3 and 4). This demonstrates that the Fas-signaling pathway, but not mitochondria-dependent pathway, is associated with the increased cas-pase-3 level that may indicate apoptosis. Additionally, because of the activation of the Fas-signaling apoptotic pathway, to contend with cell death, cardiac IGF-1 survival signaling was also increased (Figure 5) in SHS-exposed rat hearts. Furthermore, it is notable that SHS-induced dose-dependent responses of heart weight loss and interstitial fibrosis are not demonstrated in that of other cardiac gene/protein levels. The results of Masson trichrome staining showed minor to moderate fibrosis in low- and medium-dose groups. However, more severe fibrosis responses were observed in the high-dose group (Figure 1C). We conjecture that apoptotic enzyme actions are still under control in low- and medium-dose groups. However, cells severely damaged by high doses may be too weak to produce enough enzymes. This makes the gene/protein level alteration fail in a dose-dependent manner in SHS-exposed hearts.

The elevated level of the proapoptotic protein Bad was also observed in SHS-treated rat hearts (Figure C,D). Based on the observation of the 2.3-fold enhancement of BCl_2_ promoter activity by IGF-1 (Pugazhenthi et al. 1999), it is possible that the promoted IGF-1 signaling resulted in the activation of the antiapoptotic protein BCl_2_, which might counterbalance the action of elevated pro-apoptotic protein Bad and prohibit the release of cytochrome *c* from the mitochondria. Another important study (Makin et al. 2001) reported that the induction of apoptosis by Bax, whose exertions are promoted by the translocation of Bad to mitochondria and can cause cytochrome *c* release, may limit the process of the mitochondria-dependent apoptotic pathway in some cancer cells. Makin et al. (2001) conjectured that some events besides Bax oligomerization/complex formation to the mitochondria surface were absent in the cells. Hence, the abnormality of Bax action in these events may also have occurred in the SHS-stimulated cardiac tissue, explaining why the elevated Bad level did not result in cytochrome*c* release from mitochondria.

Our findings on the SHS effects in cardiac tissue include reduction of weight, alteration of morphology, possible progression of remodeling, fibrosis, and the apoptosis-related effects predicted by the activation of caspase-3 and Fas death-receptor-pathway–dependent signaling. They may be, at least partially, the possible molecular mechanisms behind how exposure of nonsmokers to SHS leads to the increased risk of cardiac events reported in epidemiologic studies. Particularly, because cardiomyocyte apoptosis and/or fibrosis is a more typical end-stage condition, it is more beneficial to alleviate these problems before the end-stage is reached. Therefore, it would seem appropriate to block cardiac Fas signaling when considering possible agents that might be helpful to control the development of apoptosis and/or fibrosis-related cardiac disease induced by SHS.

## Correction

In the manuscript originally published online, there were errors in authors’ names and affiliations; they have been corrected here.
